# Switchable Photoresponse Mechanisms Implemented in Single van der Waals Semiconductor/Metal Heterostructure

**DOI:** 10.1021/acsnano.1c07661

**Published:** 2022-01-05

**Authors:** Mingde Du, Xiaoqi Cui, Hoon Hahn Yoon, Susobhan Das, MD Gius Uddin, Luojun Du, Diao Li, Zhipei Sun

**Affiliations:** † Department of Electronics and Nanoengineering, Aalto University, Espoo FI-02150, Finland; ‡ QTF Centre of Excellence, Department of Applied Physics, Aalto University, Espoo FI-00076, Finland

**Keywords:** van der Waals heterostructure, InSe, NbTe_2_, two-dimensional metallic materials, photodetection, optical logic gate

## Abstract

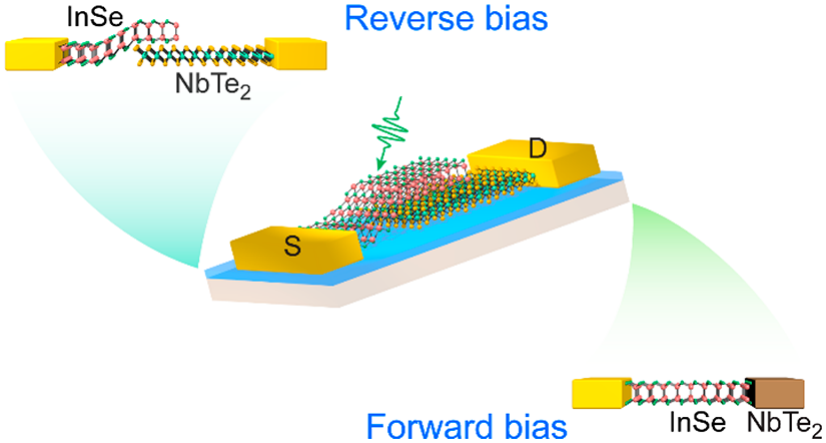

van der Waals (vdW) heterostructures based on two-dimensional (2D) semiconducting materials have been extensively studied for functional applications, and most of the reported devices work with sole mechanism. The emerging metallic 2D materials provide us new options for building functional vdW heterostructures via rational band engineering design. Here, we investigate the vdW semiconductor/metal heterostructure built with 2D semiconducting InSe and metallic 1T-phase NbTe_2_, whose electron affinity *χ*_InSe_ and work function *Φ*_NbTe_2__ almost exactly align. Electrical characterization verifies exceptional diode-like rectification ratio of >10^3^ for the InSe/NbTe_2_ heterostructure device. Further photocurrent mappings reveal the switchable photoresponse mechanisms of this heterostructure or, in other words, the alternative roles that metallic NbTe_2_ plays. Specifically, this heterostructure device works in a photovoltaic manner under reverse bias, whereas it turns to phototransistor with InSe channel and NbTe_2_ electrode under high forward bias. The switchable photoresponse mechanisms originate from the band alignment at the interface, where the band bending could be readily adjusted by the bias voltage. In addition, a conceptual optoelectronic logic gate is proposed based on the exclusive working mechanisms. Finally, the photodetection performance of this heterostructure is represented by an ultrahigh responsivity of ∼84 A/W to 532 nm laser. Our results demonstrate the valuable application of 2D metals in functional devices, as well as the potential of implementing photovoltaic device and phototransistor with single vdW heterostructure.

Two-dimensional (2D) materials, including graphene,^1^ black phosphorus,^2,3^ transition metal dichalcogenides (TMDs), and their heterostructures,^4−8^ have been extensively studied for optical and optoelectronic applications.^9−13^ They possess bandgap ranging from 0 eV to over 2 eV,^14−17^ which depends on not only the chemical composition but also the thickness of 2D flakes. The widely tunable bandgap gives rise to functional electronic devices like field effect transistors (FETs)^18,19^ and facilitates ultrasensitive detection of the light ranging from visible to near-infrared.^20−22^ Beyond single materials, many van der Waals (vdW) heterostructures integrating various materials have been successfully constructed by means of vdW stacking or chemical synthesis,^23−26^ as ultraclean surfaces and edges are readily available with 2D materials.^27^ In order to accomplish tailored functions and working mechanisms, the heterostructure devices have to be rationally designed based on band engineering.^28−30^ Accordingly, it is worth exploiting more candidate materials with distinctive band structures.

Beyond the 2D semiconducting materials with deterministic electron affinity *χ* and bandgap *E*_g_, the emerging 2D metallic materials, which have solely work function *Φ* that needs to be considered for band engineering design,^31,32^ provide new options for the development of powerful devices. Specifically, group VB metal tellurides (XTe_2_, X = V, Nb, Ta) have been theoretically calculated to be metallic and experimentally obtained by chemical synthesis.^33−36^ Quantitatively, ultrahigh electrical conductivity on the level of 10^6^ S/m was achieved with the synthesized XTe_2_ flakes.^37^ Owing to the high conductivity, NbTe_2_ was employed as conductive electrodes of WSe_2_ FET, resulting in lower contact resistance and higher carrier mobility compared with the counterpart using Cr/Au electrodes.^35^ In contrast, the chemically synthesized lateral WS_2_/NbS_2_ (semiconductor/metal) heterostructure exhibits considerable rectifying effect,^38,39^ which is a typical characteristic of diode-like heterojunction devices. The distinct transport behaviors of these vdW semiconductor/metal heterostructures are partly a result of the specific band alignment at the interfaces. Accordingly, it is promising to implement versatile functionalities at certain vdW semiconductor/metal interfaces designed based on rational band engineering.

Here, vertically stacked vdW semiconductor/metal heterostructure is designed and constructed by stacking mechanically exfoliated flakes of semiconducting InSe and metallic 1T-phase NbTe_2_. The electron affinity of thick InSe flake is extremely close to the work function of 1T-phase NbTe_2_, leading to exotic band alignment at the interface. The electrical and optoelectronic characterization, especially the photocurrent mappings, indicate that the vdW InSe/NbTe_2_ interface is switchable between heterojunction and ohmic contact, when the energy barrier at the interface is electrically tuned by bias voltage. Working as a semiconductor/metal heterojunction, it demonstrates exceptional photovoltaic effect with ∼0.41 V open-circuit voltage (*V*_OC_) and ∼380 nA short-circuit current (*I*_SC_) under 100 μW illumination of 532 nm laser. Switched to InSe phototransistor with ohmic contact to NbTe_2_ electrode, high photoresponsivity of ∼84 A/W is achieved under 10 nW illumination. These results reveal the versatile roles that 2D metallic materials could play in future optoelectronic applications.

## Results and Discussion

**Figure 1:**
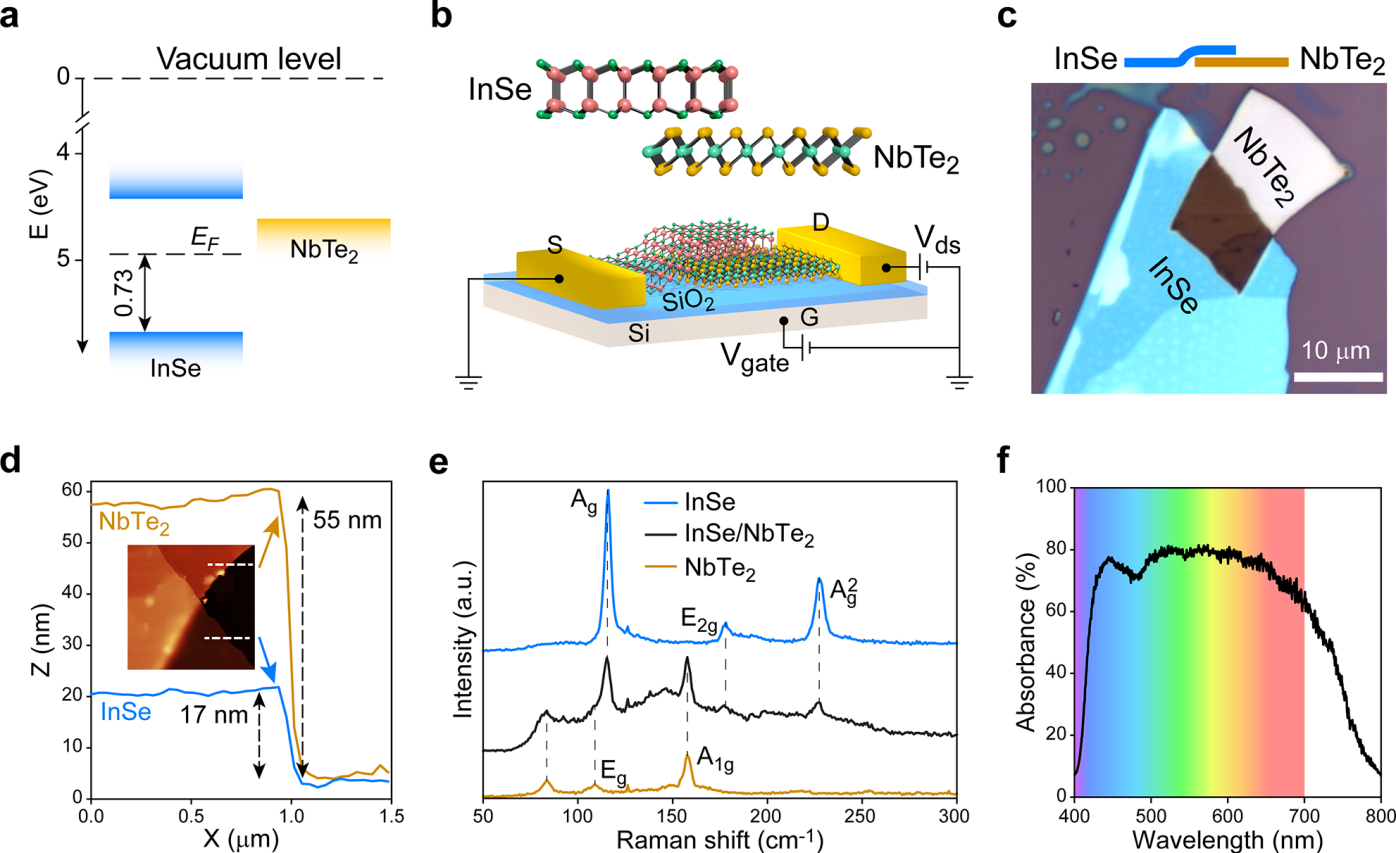
Design and characterization of the InSe/NbTe_2_ heterostructure. (a) Band diagram between the bulk materials of semiconducting InSe and metallic 1T-phase NbTe_2_. The key features are determined with the results of UPS measurements shown in Figure S1. *E*_F_ denotes the Fermi level of pristine bulk InSe, indicating that it is n-doped. (b) Schematic of the InSe/NbTe_2_ heterostructure device. The upper panel illustrates the crystal structures of InSe and NbTe_2_. The Al_2_O_3_ layer for protection is not shown in the schematic. (c) Optical microscope image of the stacked InSe/NbTe_2_ heterostructure. The top diagram illustrates the stacking order. (d) AFM characterization of the InSe/NbTe_2_ heterostructure. (e) Raman spectra of InSe, NbTe_2_, and InSe/NbTe_2_ heterostructure. (f) Absorbance of InSe/NbTe_2_ heterostructure.

In this study, InSe and NbTe_2_ are selected because of their band structures. According to the previously published experimental results, thick InSe flakes have a direct bandgap of ∼1.25 eV and electron affinity of ∼4.45 eV,^17,40^ which is extremely close to the work function of metallic 1T-phase NbTe_2_.^41^ The actual band structures of the two materials are measured with ultraviolet photoelectron spectroscopy (UPS), and the results are demonstrated in Figure S1. Accordingly, the energy bands of semiconducting InSe and metallic NbTe_2_ align as the illustration in Figure 1a. Owing to the proximity between the electron affinity of InSe and the work function of NbTe_2_, the band bending at this semiconductor/metal interface is expected to be highly adjustable. To experimentally investigate the InSe/NbTe_2_ heterostructure, a FET device was constructed on SiO_2_/Si substrate, as illustrated in the schematic of Figure 1b. In addition, an Al_2_O_3_ layer was deposited for protection of the device. The 300 nm thick SiO_2_ layer works as a dielectric layer, and the doped Si (G) on backside is used for applying gate voltage *V*_gate_. Two Ti/Au electrodes deposited on InSe and NbTe_2_ are the source (S) and drain (D) electrodes, respectively. In all the following measurements, the source electrode was grounded, and bias voltage *V*_ds_ was applied via the drain electrode.

In practice, the fabrication of InSe/NbTe_2_ heterostructure device started with mechanical exfoliation and vdW stacking of the 2D flakes (the details are described in Methods).^6,42^ Briefly, bulk 1T-phase NbTe_2_ was first exfoliated, and the obtained flakes were transferred to SiO_2_/Si substrate, followed by transferring exfoliated InSe flakes on top. As InSe has a direct bandgap only for thick flakes and NbTe_2_ is difficult to be thinned,^17^ both InSe and NbTe_2_ flakes in relatively thick form were used. Optical microscope images of the InSe/NbTe_2_ heterostructure and fabricated FET device are shown in Figure 1c and Figure S2, and the overlapping region has an area of ∼8 μm × 10 μm. Thickness of the flakes was measured by atomic force microscopy (AFM), and the line profiles in Figure 1d indicate that the InSe and NbTe_2_ flakes are ∼17 nm and ∼55 nm thick, respectively. The two flakes were further identified by Raman spectra. As shown in Figure 1e, Raman spectra were collected at both the single materials and the heterostructure. In the Raman spectrum of InSe, three peaks are observed at ∼116 cm^–1^, ∼178 cm^–1^, and 227 cm^–1^, attributed to A_g_, E_2g_, and A_g_^2^ phonon modes, respectively.^43,44^ The Raman spectrum of NbTe_2_ flake consists of three prominent peaks (∼84 cm^–1^, ∼109 cm^–1^, and ∼158 cm^–1^) and two weak peaks (∼219 cm^–1^ and ∼254 cm^–1^, detailed in Figure S3). These results agree well with the published results of NbTe_2_ flakes grown by chemical vapor deposition and vapor phase transport.^36,45^ All the major peaks described above could be found in the Raman spectrum of InSe/NbTe_2_ heterostructure, indicating the high quality of fabricated vdW heterostructure. Furthermore, photoluminescence (PL) spectra of the flakes were measured, and the results are depicted in Figure S4. The obvious peak centered at photon energy of around 1.25 eV is a typical value of thick InSe flakes,^17^ while the metallic NbTe_2_ does not show any PL peak. The significant darkness of InSe/NbTe_2_ heterostructure in Figure 1c is unexpected, and it is generally observed in other samples as well (Figure S5a–d). To quantitatively comprehend this observation, the reflection of these 2D materials on SiO_2_/Si substrate was measured, and the absorbance was calculated with a silver mirror as reference (Figure S5e). According to the calculated absorbance demonstrated in Figure S5f, the InSe and NbTe_2_ flakes mainly absorb light in the wavelength ranges of ∼600–700 nm and ∼450–600 nm, respectively. It means that InSe and NbTe_2_ are complementary for the absorption of visible light. Consequently, the absorbance of InSe/NbTe_2_ heterostructure is universally enhanced in the whole visible range (up to ∼80% at ∼500–550 nm), as depicted in Figure 1f. One reasonable explanation for the high absorbance is the joint absorption effect of the two thick flakes, as observed in another case of thick InSe/Te (40 nm/120 nm) heterostructure, whose overlapping area is significantly dark as well.^46^ However, to the best of our knowledge, dark vdW heterostructures are rarely observed, even in the ones built with thick flakes. Therefore, this phenomenon is worth investigating by systematic and meticulous spectroscopic characterization. Despite the unknown cause, the high absorbance is expected to benefit the photodetection with InSe/NbTe_2_ heterostructure.

The working mechanism of the InSe/NbTe_2_ heterostructure device could be predicted based on the published works,^33,36,38^ where 2D semiconductor/metal heterostructure devices with various materials exhibit distinctive transport behaviors. On the basis of computational study,^33^ the work function of metallic NbS_2_ drops in the bandgap of WS_2_, whereas the work function of metallic NbTe_2_ is much lower than the valence band maximum of WSe_2_. As a result, an energy barrier with considerable height emerges because of band bending at the WS_2_/NbS_2_ interface, while it is absent at the WSe_2_/NbTe_2_ interface. These band diagrams lead to an apparent rectifying effect in heterogeneous WS_2_/NbS_2_ device and remarkably low contact resistance of homogeneous WSe_2_ FET with NbTe_2_ electrodes.^35,39^ On the basis of these results, the InSe/NbTe_2_ interface is predicted to be switchable between heterojunction and ohmic contact, as the difference between the electron affinity of InSe and the work function of NbTe_2_ is so little.

**Figure 2:**
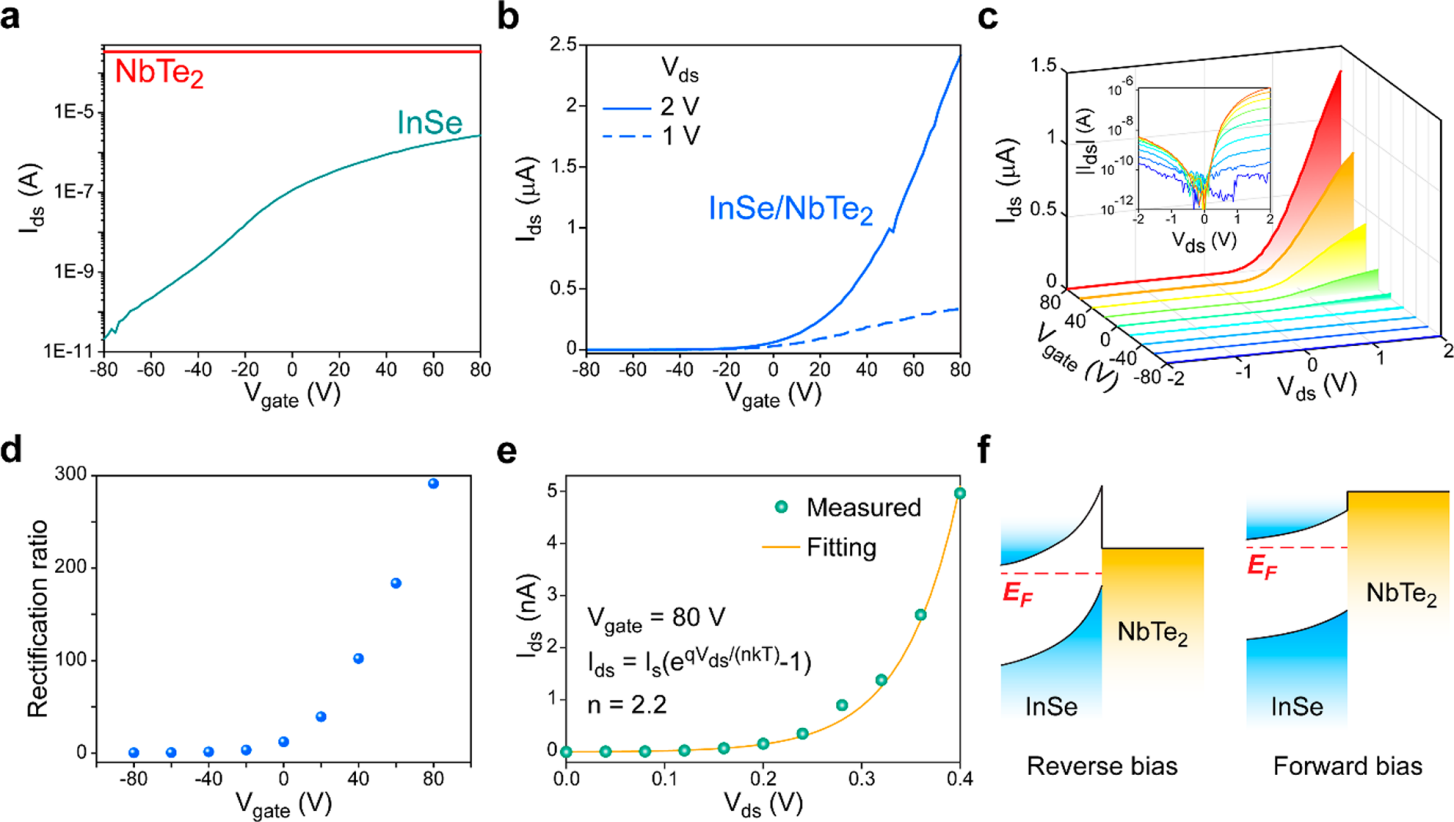
Electrical characterization of the heterostructure device. (a) Transfer curves of the devices with pure InSe or NbTe_2_ channel. Bias voltages *V*_ds_ of 2 and 0.1 V were applied in the measurements of InSe and NbTe_2_ devices, respectively. (b) Transfer curves of the InSe/NbTe_2_ heterostructure device measured with bias voltage *V*_ds_ of 2 V and 1 V. (c) Gate voltage dependent output *I*_ds_–*V*_ds_ curves of the InSe/NbTe_2_ heterostructure device. The inset shows |*I*_ds_| on a logarithmic scale. (d) Gate voltage dependent rectification ratio calculated with the results in (c). (e) Fitting of the output *I*_ds_–*V*_ds_ curve measured at *V*_gate_ = 80 V by Shockley diode function.^47^ Ideality factor of *n* = 2.2 is extracted from the fitting. (f) Schematic of band bending at the interface between n-doped InSe and metallic NbTe_2_ under reverse and forward biases when a positive gate voltage is applied. *E*_F_ denotes the Fermi level of InSe.

Initially, electrical characterization was conducted to reveal the basic working mechanism of InSe/NbTe_2_ heterostructure, and the results are depicted in Figure 2. For comparison, the FETs with pure InSe or NbTe_2_ channel were fabricated and measured as well. The transfer curves shown in Figure 2a, as well as the gate dependent output *I*_ds_*–V*_ds_ curves in Figure S6, suggest that the exfoliated InSe flake is intrinsically n-doped and highly conductive when it is heavily doped by positive gate voltage, and the NbTe_2_ flake is a metal with conductivity independent of gate voltage. In addition, the linear output *I*_ds_*–V*_ds_ curves indicate ohmic contact between the flakes and Ti/Au electrodes. These results agree well with the published results of semiconducting InSe and metallic NbTe_2_, indicating the high quality of exfoliated InSe and NbTe_2_ flakes. In the following, the InSe/NbTe_2_ heterostructure FET was measured with the configuration illustrated in Figure 1b, and two different bias voltages *V*_ds_ of 2 V and 1 V were applied. As shown in Figure 2b and Figure S7, the *I*_ds_ could be considerably modulated by gate voltage, leading to a high current On/Off ratio of nearly 10^5^. The high On current and On/Off ratio were maintained even 2 months after device fabrication (Figure S8). In contrast, the bare device without protective Al_2_O_3_ layer shows low On/Off ratio of <10^3^ when it was measured right after fabrication (Figure S9). This comparison clearly indicates the protection effect of Al_2_O_3_ layer. Besides, the significantly nonlinear relation between the *I*_ds_ and *V*_ds_ at positive gate voltage verifies the existence of Schottky barrier at the InSe/NbTe_2_ interface. The Schottky barrier could be further confirmed with the output *I*_ds_*–V*_ds_ curves in Figure 2c, which exhibit a common diode-like rectifying effect. The *I*_ds_ is significantly suppressed when a negative *V*_ds_ is applied, as the reverse bias increases the height of Schottky barrier. The rectifying effect could be quantitatively assessed by rectification ratio calculated with the *I*_ds_ at *V*_ds_ = 2 V and *V*_ds_ = −2 V. As depicted in Figure 2d, the rectification ratio rises to ∼300 when the gate voltage of *V*_gate_ = 80 V is applied, and a higher value of >10^3^ is obtained with another device (Figure S10). Furthermore, the output curve measured at *V*_gate_ = 80 V is fitted by the Shockley diode function and shown in Figure 2e,^47^ leading to an ideality factor of *n* = 2.2.

The exceptional rectifying effect could be readily explained with the band diagram illustrated in Figure 2f. When a negative reverse bias is applied to NbTe_2_, its Fermi level is lifted relative to InSe, resulting in an increased band mismatch according to Figure 1a. The band mismatch induces significant band bending on InSe side, and a relatively high Schottky barrier is built at the InSe/NbTe_2_ interface. Therefore, the *I*_ds_ through InSe/NbTe_2_ heterojunction is dominated by tunneling current. Once a high positive forward bias (e.g., *V*_ds_ = 2 V) is applied, the Fermi level of NbTe_2_ is considerably lowered; thus the band bending on InSe side is eliminated. In this case, the majority carriers in InSe could easily diffuse to NbTe_2_ and lead to a high conductance of the heterostructure channel. Actually, this band bending conflicts with the band diagram determined with UPS (Figure 1a), suggesting that significant n-doping of semiconducting InSe is induced in the device fabrication process, especially the deposition of Al_2_O_3_ (see Methods), where 2 nm thick aluminum layer was deposited and oxidized as seeding layer. The strongly adjustable band diagram at InSe/NbTe_2_ interface makes it distinct from the massively investigated vdW heterostructures built with semiconductors. This could be verified with the following optoelectronic measurements.

**Figure 3:**
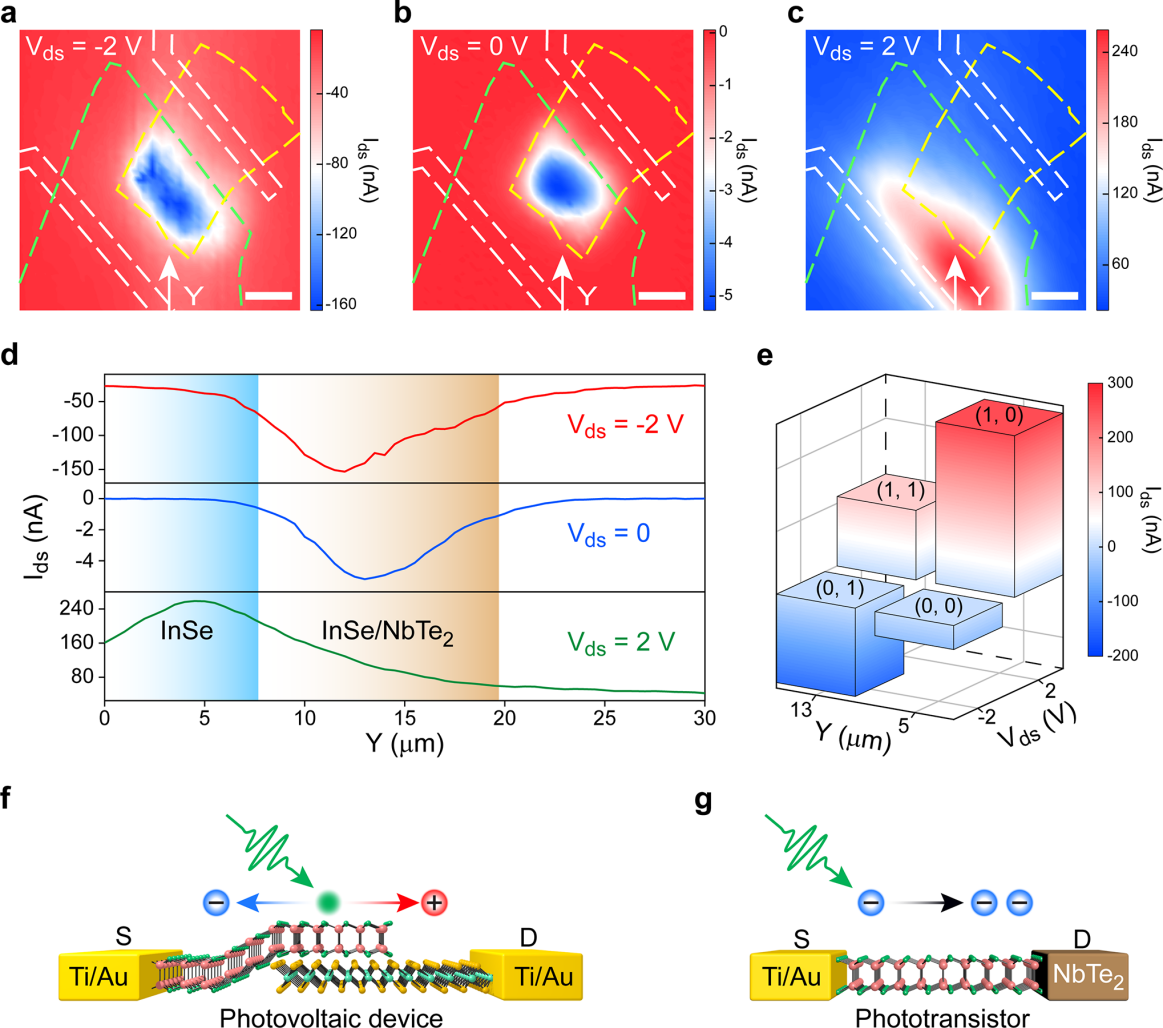
Switchable photoresponse mechanisms of InSe/NbTe_2_ heterostructure. (a–c) Photocurrent mappings in InSe/NbTe_2_ heterostructure device at various bias voltages *V*_ds_ of −2, 0, and 2 V. The green, yellow, and white dashed lines illustrate the outlines of InSe, NbTe_2_, and Ti/Au electrodes, respectively. Scale bars, 5 μm. (d) Line scannings extracted from the mappings at the positions and directions indicated by white arrows in (a)–(c). The blue and brown shades indicate the *Y* positions of pure InSe and InSe/NbTe_2_ heterostructure. (e) Truth table of the conceptual XOR logic gate with inputs of laser beam position (*Y*) and bias voltage (*V*_ds_) and output of *I*_ds_. (f, g) Schematic of the InSe/NbTe_2_ heterostructure device switched between the photovoltaic device (f, reverse bias) and phototransistor (g, forward bias). The role of NbTe_2_ is switched between a heterojunction component and a contact electrode.

Photocurrent mappings are performed to achieve a more profound interpretation of the working mechanisms of InSe/NbTe_2_ heterostructure device. In order to study the intrinsic heterostructure, the gate voltage was fixed at *V*_gate_ = 0 V in the measurements. The device was laterally moved with steps of 0.5 μm, and a 532 nm laser beam with 1 μW power was focused on the plane of heterostructure and swept in the area shown in Figure S2. Source-drain current *I*_ds_ (measured with the same configuration in Figure 1b) was recorded under various bias voltages *V*_ds_ of −2 V, 0 V, and 2 V, and the results are depicted in Figure 3a–c. The green, yellow, and white dashed lines in Figure 3a–c outline the positions of InSe, NbTe_2_, and Ti/Au electrodes. An intuitive observation of the mappings is the bias-dependent positions of peak *I*_ds_. The peak *I*_ds_ is obtained at InSe/NbTe_2_ stacking area under bias voltage *V*_ds_ of −2 V and 0 V. In contrast, under high forward bias voltage of *V*_ds_ = 2 V, the peak *I*_ds_ is obtained at pure InSe area. This observation could be more clearly and quantitatively illustrated by the line scannings extracted at the white arrows in Figure 3a–c, and the results are shown in Figure 3d. On the basis of bias-dependent peak *I*_ds_ positions, a conceptual XOR logic gate with truth table summarized in Figure 3e is proposed. Two inputs of this logic gate are *Y* position of the laser beam (e.g., “0” for *Y* = 5 μm, “1” for *Y* = 13 μm) and bias voltage *V*_ds_ (e.g., “0” for *V*_ds_ = −2 V, “1” for *V*_ds_ = +2 V) in Figure 3d, and the output is source−drain current *I*_ds_ (e.g., “0” for small |*I*_ds_| and “1” for large |*I*_ds_|). The bias dependent photocurrent generation is also confirmed with the device shown in the inset of Figure S10. The irregular outline of InSe/NbTe_2_ overlapping area makes it easy to identify the photoresponse area in the whole heterostructure (Figure S11a–c). The mappings of photocurrent under high negative gate voltage (Figure S11d–f) suggest that this phenomenon occurs even when the Fermi level of InSe channel is significantly lowered by negative *V*_gate_, despite the decreased absolute photocurrent. On the basis of the results in Figure S10, this device demonstrates a weak rectifying effect under negative gate voltage of *V*_gate_ = −80 V. Therefore, the bias dependent switchable photoresponse mechanisms exist as long as the device shows a rectifying effect or an effective Schottky barrier exists at the heterostructure interface.

The bias dependent peak photocurrent positions imply switchable photodetection mechanisms of InSe/NbTe_2_ heterostructure, attributed to the band bending tuned by bias voltage *V*_ds_. Essentially, the excited photocarriers are mainly generated in InSe. Under reverse bias, a high Schottky barrier is built, and the device turns to heterojunction (Figure 2f). Thus, the heterostructure responds to light illumination as a photovoltaic device (Figure 3f). In other words, only the electron–hole pairs generated at InSe/NbTe_2_ stacking area could be efficiently separated by the built-in electric field and contribute to photocurrent (Figure 3a). However, the energy barrier is significantly lowered or even vanishes when a large forward bias is applied (Figure 2f). Consequently, the device works as a phototransistor with homogeneous InSe channel and NbTe_2_ conductive electrode (Figure 3g), and the thick InSe channel with direct bandgap could absorb light illumination with high efficiency.^17^ Given the above, the InSe/NbTe_2_ heterostructure device could be readily switched between heterojunction photovoltaic device and InSe phototransistor with NbTe_2_ contact electrode, indicating the rationality of band engineering design in Figure 1a.

**Figure 4:**
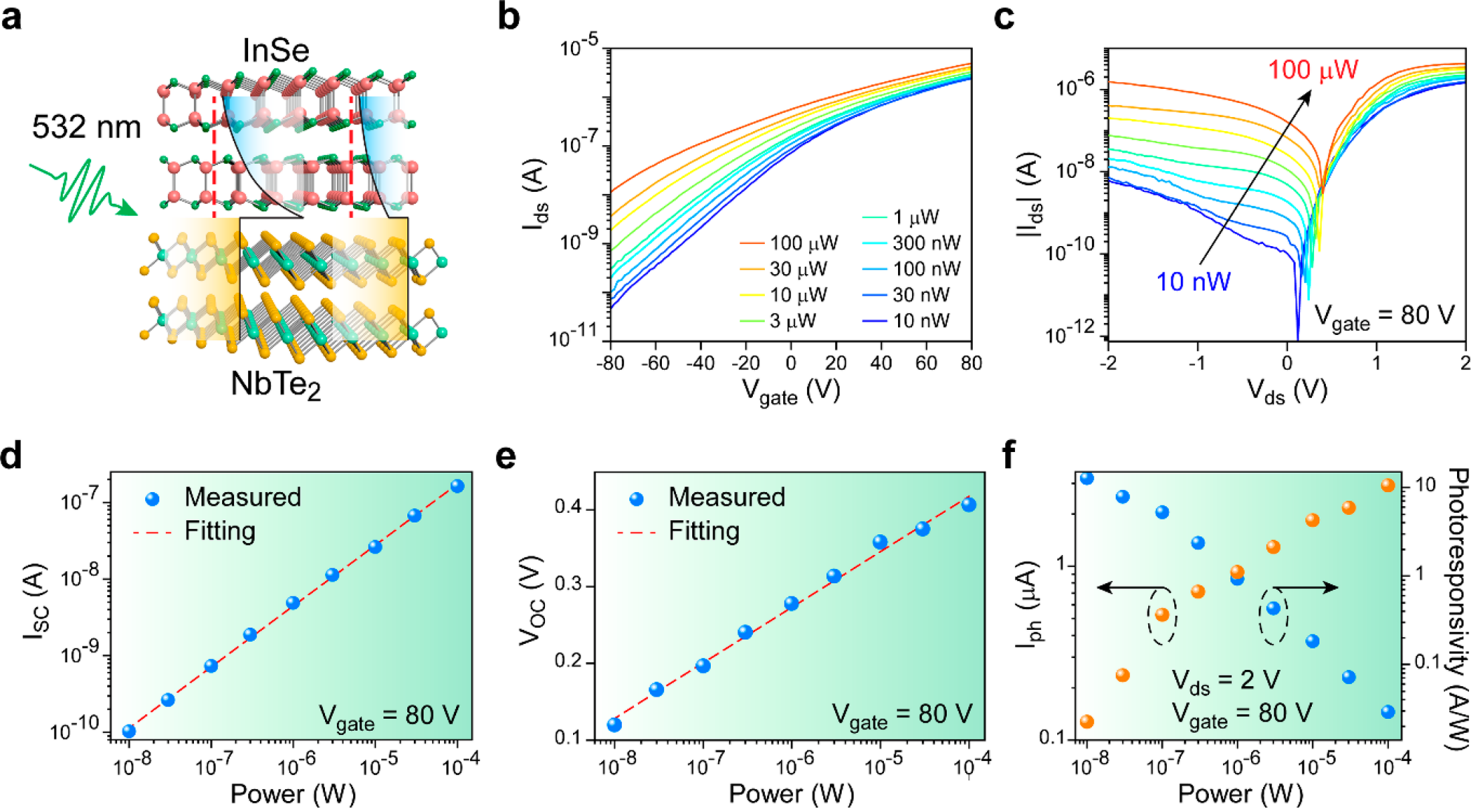
Overall photodetection performance of InSe/NbTe_2_ heterostructure device. (a) Schematic of the switchable InSe/NbTe_2_ heterostructure for the detection of 532 nm laser. Working mechanism of this device depends on bias-dependent band bending at the interface. (b) Transfer curves of InSe/NbTe_2_ phototransistor illuminated by 532 nm laser beam with gradient power. (c) *I*_ds_*–V*_ds_ curves of the InSe/NbTe_2_ heterostructure device under light illumination. (d, e) Short-circuit current *I*_SC_ (d) and open-circuit voltage *V*_OC_ (e) extracted from the *I*_ds_*-V*_ds_ curves in (c). The results are fitted by *I*_SC_ ∝ *Power^α^* and *V*_OC_ ∝ ln(*Power*). (f) Photocurrent *I*_ph_ and photoresponsivity calculated with the data in (c).

Finally, photodetection performance of the switchable InSe/NbTe_2_ heterostructure device is systemically investigated with 532 nm laser illumination. As illustrated in the schematic of Figure 4a, the photoresponse mechanism is subject to bias-dependent band bending at the interface (Figure 3f,g). For a phototransistor working under bias voltage *V*_ds_ = 2 V, *I*_ds_ is raised when the laser power increases from 10 nW to 100 μW (Figure 4b). Notably, *I*_ds_ at *V*_gate_ = −80 V increases significantly compared with the values at *V*_gate_ = 80 V. This phenomenon could be explained by the change of Fermi level *E*_F_ of InSe. *E*_F_ is significantly declined, and the density of carriers is extremely low under gate voltage of *V*_gate_ = −80 V. Thus even the photocarriers induced by a low-power illumination could lead *E*_F_ to rise considerably. Therefore *I*_ds_ changes a lot as the illumination power is increased. The carrier density is intrinsically high under gate voltage *V*_gate_ = 80 V. Thus the photocarriers finitely change the band diagram or contribute to the *I*_ds_ dominated by the thermionic current. Properties of the InSe/NbTe_2_ heterostructure working as a photovoltaic device are revealed by the *I*_ds_*–V*_ds_ curves measured under laser illumination with various power, and the results are shown in Figure 4c and Figure S12. The large *I*_SC_ (Figure 4d) and high *V*_OC_ (Figure 4e) are characteristics of a photovoltaic device. The highest *V*_OC_ in Figure 4e is ∼0.41 V, and large *I*_SC_ of ∼380 nA (Figure S13) is obtained with the device demonstrated in Figure S10, indicating the excellent performance of InSe/NbTe_2_ photovoltaic device. As demonstrated in Figure S14, the InSe/NbTe_2_ heterostructure device works with higher response speed in photovoltaic mode and lower response speed in phototransistor mode, and the ultimate response time should be less than 10 ms. Besides, the photocurrent *I*_ph_ and corresponding photoresponsivity are calculated (Figure 4f) based on the *I*_ds_*–V*_ds_ curves measured in dark condition (Figure 2c), and a high photoresponsivity of ∼12 A/W is achieved with the InSe phototransistor when a bias voltage of *V*_ds_ = 2 V is applied. All the results in Figure 4 are measured with the laser spot centered at stacking InSe/NbTe_2_ area. Yet, the highest photocurrent in the phototransistor should be obtained when the pure InSe is illuminated under positive bias, according to Figure 3c. Therefore, the photoresponse was additionally measured with 10 nW illumination centered at pure InSe, where peak *I*_ds_ is obtained in Figure 3c. On the basis of output *I*_ds_–*V*_ds_ curves demonstrated in Figure S15, a remarkably high photoresponsivity of ∼84 A/W is achieved. This photoresponsivity is superior to many of the published works of InSe-based photodetectors, as compared in Table S1. The exceptional photodetection performance affirms the rationality of the band engineering design discussed in Figure 1, indicating a promising strategy for developing powerful vdW devices. As summarized in Table S2, the performance of this InSe phototransistor with metallic NbTe_2_ electrode is comparable with or even better than the InSe devices with contact electrodes of other materials.

## Conclusions

Switchable photoresponse mechanisms have been implemented in single van der Waals semiconductor/metal heterostructure. Its working mechanism could be readily switched by the bias voltage that tunes band bending at the semiconductor/metal interface. The electrical characterization demonstrates a significant rectifying effect, indicating an inherent diode-like heterojunction. Further systematic optoelectronic measurements results indicate that reverse bias switches the heterostructure to a photovoltaic device, and alternatively, large forward bias turns it to a phototransistor with InSe channel and NbTe_2_ electrode. Overall, it exhibits exceptional electronic and optoelectronic performance of >10^3^ rectification ratio and ∼84 A/W photoresponsivity, suggesting that it is a promising candidate for practical applications. The rational band engineering design in this vdW heterostructure and its exceptional device performance indicate a promising strategy for building versatile 2D optoelectronic devices and highlight the functionality of 2D metallic materials.

## Methods

### UPS Measurements

Bulk materials of InSe and NbTe_2_ were used for UPS measurements in high vacuum. The materials were sputtered with Ar^+^ for cleaning, and a bias voltage of −10 V was applied. The photon energy of the UV light source is 21.22 eV, and the work function of the analyzer is 4.66 eV.

### Preparation and Characterization of Two-Dimensional Flakes

The InSe and NbTe_2_ flakes were mechanically exfoliated from bulk materials (2D Semiconductors). In the following, NbTe_2_ flakes were transferred to silicon substrates covered with 300 nm thick SiO_2_, and the InSe flakes supported by polydimethylsiloxane (PDMS) were stacked on top. Raman spectra were collected with WITec micro-Raman system, and a 532 nm laser was used for excitation. The reflection of silver mirror, InSe, NbTe_2_, and InSe/NbTe_2_ heterostructures was measured with SNOM system (WITec alpha300). The absorbance of the materials is calculated with the reflection of a silver mirror as reference: absorbance = [(*R*_Ag_ – *R*_X_)/*R*_Ag_] × 100%, where *R*_Ag_ and *R*_X_ are the reflection of silver mirror and 2D materials, respectively. The PL measurements were also conducted with a SNOM system, and a 532 nm laser with power of ∼1 mW was used for excitation. AFM image of the flakes was collected by Dimension Icon system (Bruker).

### Device Fabrication

The patterning and deposition of metal electrodes were accomplished through patterning PMMA photoresist with electron beam lithography (EBL, Vistec EBPG 5000), deposition of Ti/Au (5 nm/100 nm) with electron beam evaporation system (MASA IM-9912), and finally the lift-off process in acetone. For optimization, the device was annealed at 180 °C in high vacuum (∼10^–5^ mbar) for 2 h (AML-AWB wafer bonding machine). Then a 2 nm thick Al seeding layer was deposited and heated at 130 °C in the air for 2 min. Afterward, 20 nm thick Al_2_O_3_ was deposited at 120 °C by atomic layer deposition (Beneq TFS-500), followed by second annealing with the same conditions. Finally, the Ti/Au electrodes were connected to a printed circuit board (PCB) by wire bonding. The fabrication of bare device was terminated after the first annealing.

### Electrical and Optoelectronic Measurements

Keithley 2401 and Keithley 2400 were used for applying bias voltage *V*_ds_ and gate voltage *V*_gate_, and the drain-source current *I*_ds_ was measured with Keithley 2401. Data acquisition was accomplished with a customized LabVIEW program. The PCB with fabricated device was fixed on the lateral movement stage of the SNOM system, which was precisely controlled by the LabVIEW program. In the optoelectronic test, a 532 nm laser with adjustable power was illuminated on the device through a 20× objective (NA = 0.4).

## Supporting Information Available

Materials characterization with Raman, PL, UPS, and absorption spectra, output *I*_ds_–*V*_ds_ curves and transfer curves of additional devices, photocurrent mapping at negative gate voltage, short-circuit current at 100 μW illumination, measurement of response time, and comparison of various InSe-based photodetectors (PDF)
