# Supplementary material for: Switchable Photoresponse Mechanisms Implemented in Single van der Waals Semiconductor/Metal Heterostructure
Source: ACS Nano. 2022 Jan 5;16(1):568–76. doi: 10.1021/acsnano.1c07661 (PMC8793132; doi:10.1021/acsnano.1c07661)
Supplement: Supplementary file 1 — nn1c07661_si_001.pdf [file nn1c07661_si_001.pdf]

Switchable Photoresponse Mechanisms Implemented in Single van der Waals Semiconductor/Metal Heterostructure

Mingde Du[†,*]*, Xiaoqi Cui*[†]*, Hoon Hahn Yoon*[†]*, Susobhan Das*[†]*, MD Gius Uddin*[†]*, Luojun Du*[†]*, Diao Li*[†]*, Zhipei Sun*[†,‡,*]

[†]Department of Electronics and Nanoengineering, Aalto University, Espoo FI-02150, Finland

[‡]QTF Centre of Excellence, Department of Applied Physics, Aalto University, Espoo FI-00076, Finland

*Email: mingde.du@aalto.fi, zhipei.sun@aalto.fi.

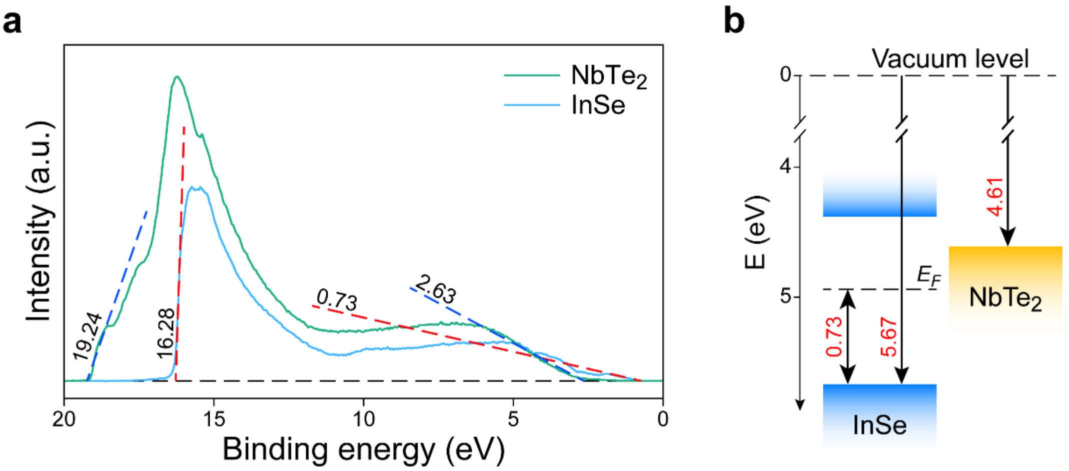

Figure S1. UPS measurement of the 2D materials. (a) UPS spectra of separate InSe and $NbTe_2$ bulk materials. The black dashed line is 0 level of intensity, and the blue and red dashed lines indicate the linear fittings for extracting the cutoffs in the spectra. Accordingly, the work function of metallic $NbTe_2$ is calculated as: ~21.22 - (19.24 - 2.63) = 4.61 eV. The valance band maximum (VBM) of InSe is calculated as: ~21.22 - (16.28 - 0.73) = 5.67 eV, and the difference between its VBM and Fermi level is ~0.73 eV. (b) Band diagram of InSe and $NbTe_2$ with the key features extracted from (a).

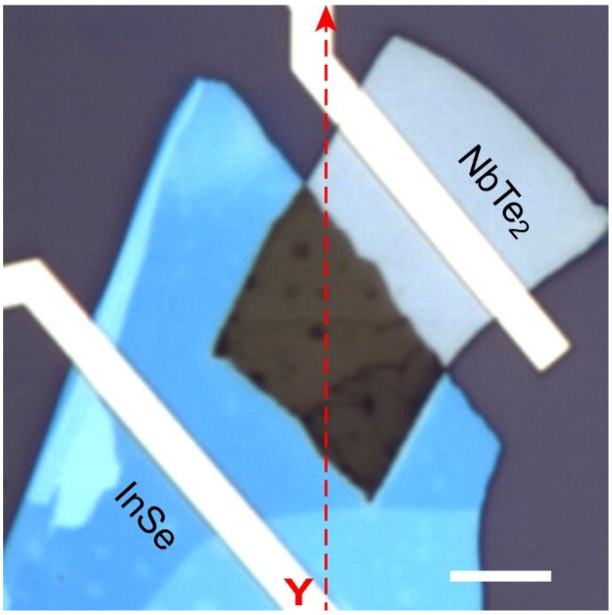

Figure S2. Optical microscope image of the fabricated InSe/$NbTe_2$ heterostructure device. The two bright stripes are Ti/Au electrodes, and the dark area at the center is overlapping InSe/$NbTe_2$ heterostructure with InSe on top. The red dashed arrow indicates the position and direction of photocurrent line scanning shown in Figure. 3d. Scale bar, 5 μm.

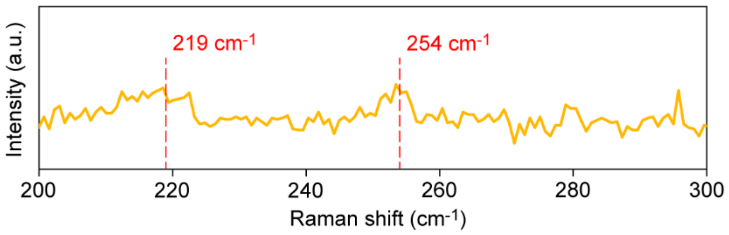

Figure S3. Raman spectrum of $NbTe_2$ flake. The two peaks at 219 cm^{-1} and 254 cm^{-1} agree well with the published results.[1]

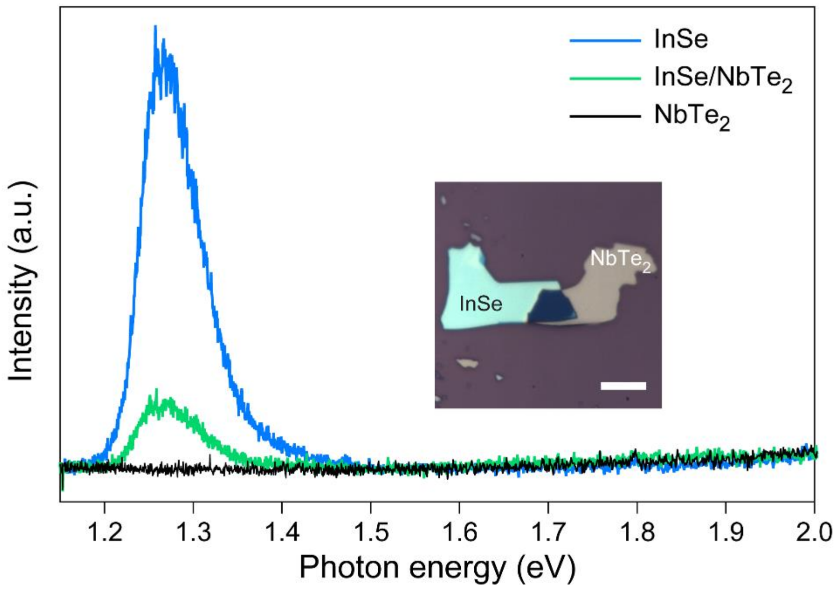

Figure S4. Photoluminescence (PL) characterization of the 2D materials. As shown in the figure, a strong PL peak centered at around 1.25 eV is detected at pure InSe flake. In contrast, the PL of InSe/$NbTe_2$ heterostructure is significantly lowered because of charge transfer.[2] As a metallic material, pure $NbTe_2$ doesn't show any noticeable PL signal. The power of excitation with 532 nm laser is ~1 mW.

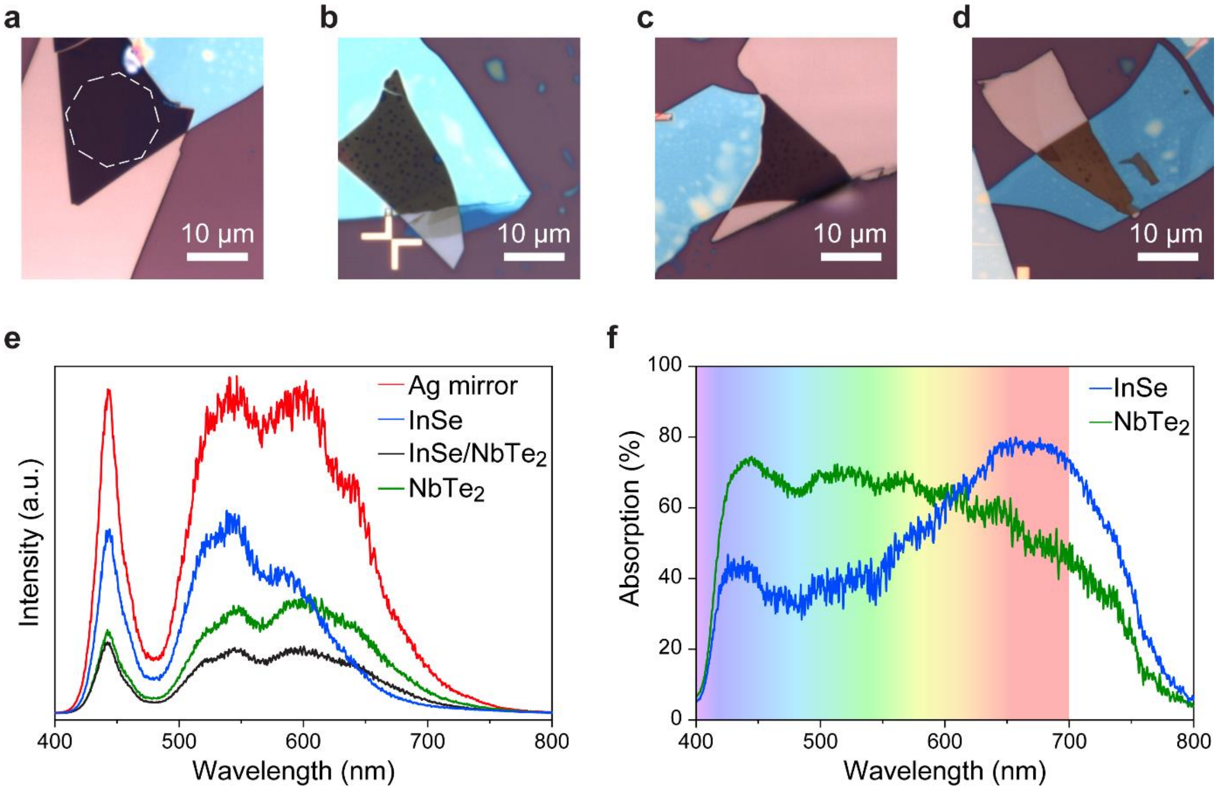

Figure S5. The absorbance of dark $InSe/NbTe_2$ heterostructures. (a-d) Additional samples of $InSe/NbTe_2$ heterostructures on SiO_2/Si substrate, the blue flakes are $NbTe_2$ on the bottom. The dashed circle in (a) indicates the size and position of the aperture that was used for measuring the reflection of $InSe/NbTe_2$ heterostructure. (e) Reflection of Ag mirror, InSe flake, $InSe/NbTe_2$ heterostructure and $NbTe_2$ flake in (a). The same white light source was used for all the measurements. (f) The absorbance of InSe and $NbTe_2$ flakes. The absorbance spectra are calculated with the data in (e): absorbance = $(R_{Ag} - R_X)/R_{Ag}*100$ %, where R_{Ag} and R_X are the reflection of Ag mirror and 2D materials, respectively.

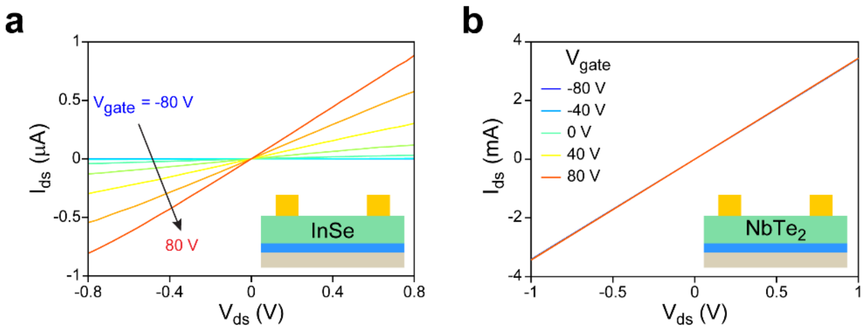

Figure S6. Gate dependent output I_{ds}-V_{ds} curves of the FETs with pure InSe or $NbTe_2$ channel. The linear curves indicate excellent contact between the Ti/Au electrodes and 2D flakes.

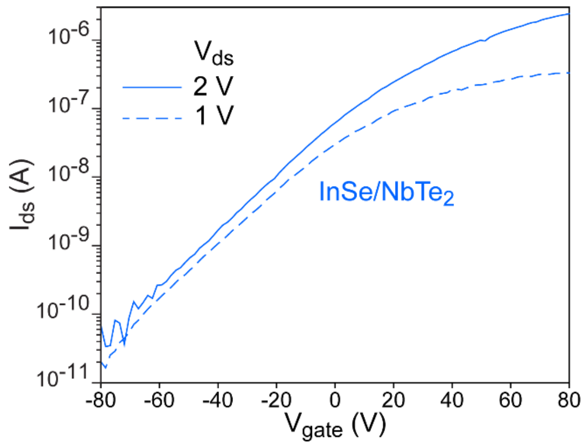

Figure S7. Transfer curves of the $InSe/NbTe_2$ heterostructure device in dark condition.

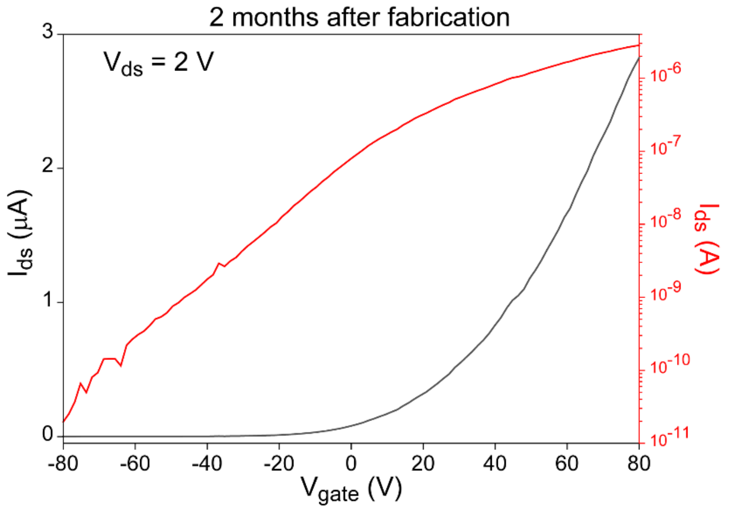

Figure S8. Transfer curve of the $InSe/NbTe_2$ heterostructure device measured 2 months after fabrication. The result indicates that this device is exceptionally stable.

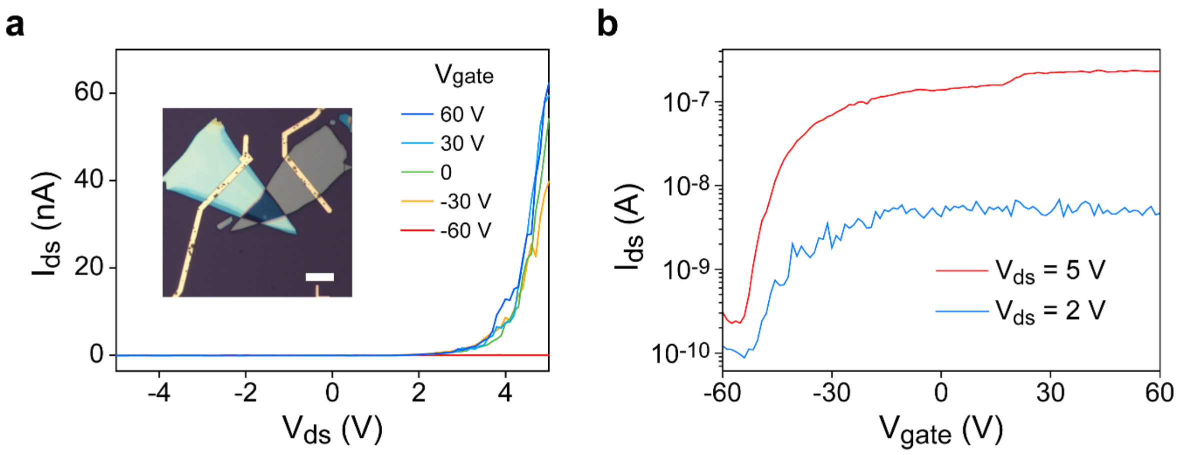

Figure S9. I_{ds}-V_{ds} curves (a) and transfer curves (b) of a bare InSe/$NbTe_2$ heterostructure device without Al_2O_3 protection layer. The bare device has a size comparable with the devices with an Al_2O_3 protection layer, and was measured in the air on the next day after fabrication. Similarly, the InSe side is grounded and bias voltage V_{ds} is applied on the $NbTe_2$ side. The I_{ds}-V_{ds} curves (a) at various V_{gate} show a significant diode-like rectifying effect, while the threshold voltage at ~2 V is much higher than ~0.4 V for the case of Al_2O_3 protected devices. On the other hand, I_{ds} at On state in the transfer curves (b) is lower than 1 μA, which is a typical value for the protected devices. The inset of (a) is an optical image of this bare device. Scale bar, 10 μm.

It is worth noting that the results of bare reference device were collected on the next day after fabrication, I_{ds} is even lower than the results of the protected device measured 2 months after fabrication (Figure S8). Obviously, the bare device is InSe/$NbTe_2$ heterostructure unstable in the air.

Overall, the bare InSe/$NbTe_2$ heterostructure device shows a diode-like rectifying effect and gate modulation effect. However, the quantitative performance is not comparable with the device protected with Al_2O_3 layer.

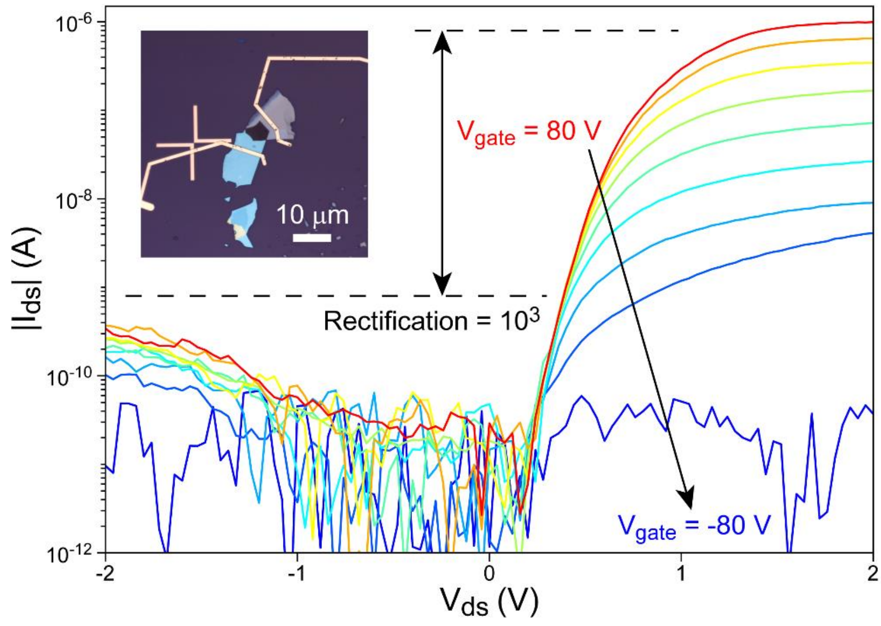

Figure S10. Output I_{ds}-V_{ds} curves of another InSe/$NbTe_2$ heterostructure device measured in dark condition in the air. A rectification ratio of >10^3 is obtained.

This device shows a significant rectifying effect under -60 V or higher gate voltage. Under gate voltage of V_{gate} = -80 V, I_{ds} significantly fluctuates when negative V_{ds} is applied and has a value robustly higher than 10^{-11} A when positive V_{ds} is applied. Therefore, it's reasonable to conclude that a weak rectifying effect exists under high negative gate voltage of V_{gate} = -80 V.

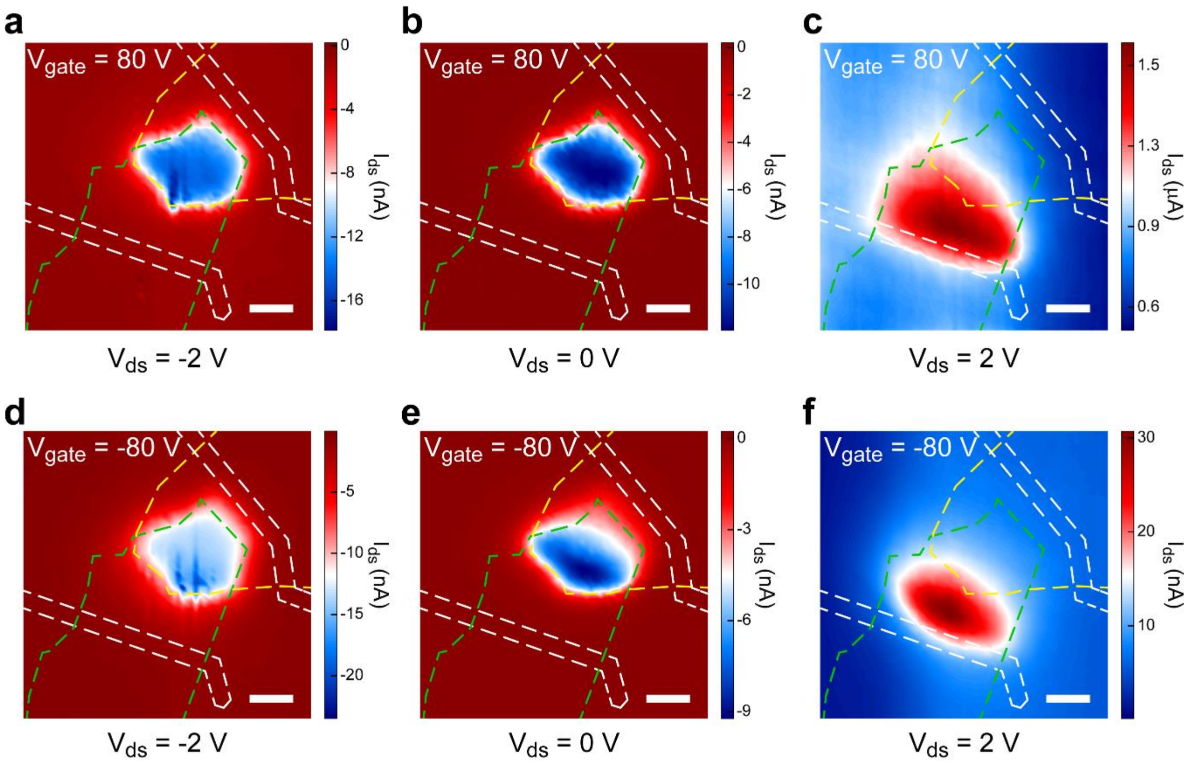

Figure S11. Bias-dependent photocurrent mapping of another device. (a-c) Photocurrent mappings at V_{gate} = 80 V. (d-f) Photocurrent mappings at V_{gate} = -80 V. The optical microscope image of this device is shown in the inset of Figure S10. The white, orange, and green dashed lines outline the Ti/Au electrodes, $NbTe_2$ flake and InSe flake. Scale bars, 5 µm.

The irregular shape of InSe/$NbTe_2$ stacking area makes it easy to identify the position laser illumination when high photocurrent is obtained.

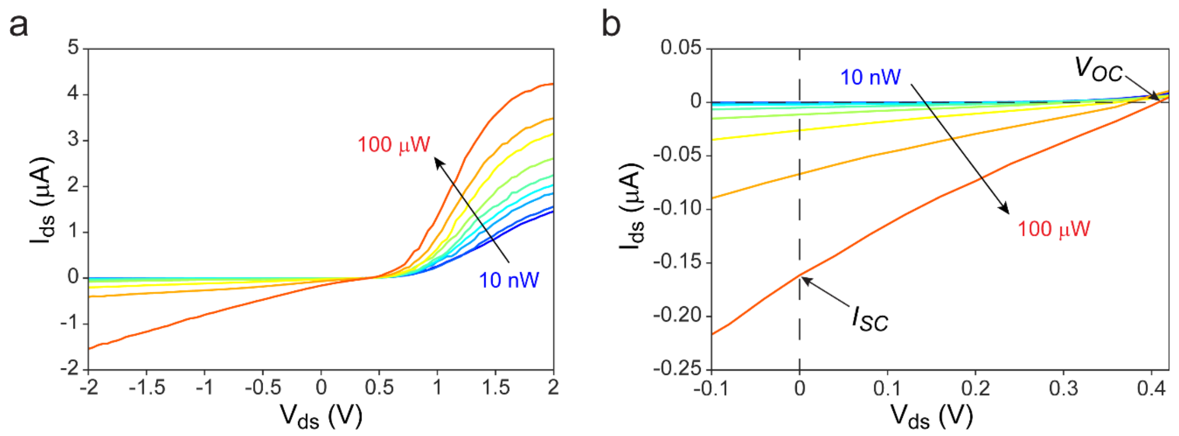

Figure S12. (a) I_{ds}-V_{ds} curves of InSe/$NbTe_2$ heterostructure device under the illumination of 532 nm laser with power ranging from 10 nW to 100 µW. Gate voltage of V_{gate} = 80 V was applied in the measurements. (b) Short-circuit current I_{SC} and open-circuit voltage V_{OC} extracted from the I_{ds}-V_{ds} curves.

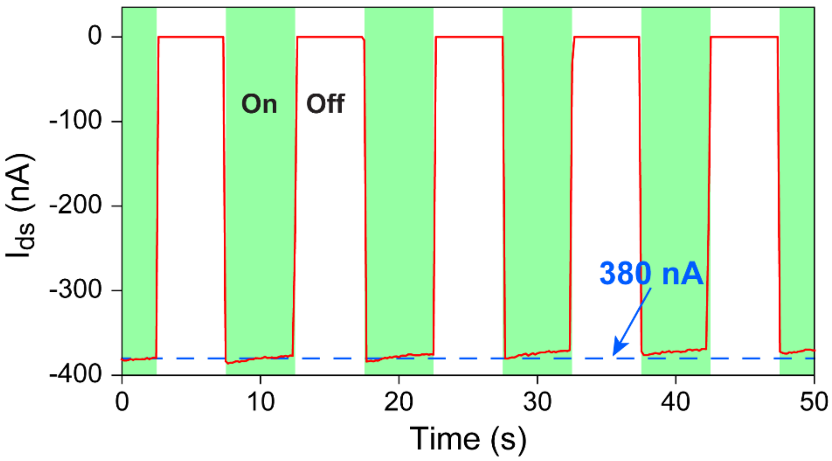

Figure S13. Short-circuit current I_{sc} under 100 μW illumination of the 532 nm laser. In this test, a gate voltage of V_{gate} = 80 V and a bias voltage of V_{ds} = 0 V are applied. The laser spot was focused at InSe/$NbTe_2$ stacking area, and the illumination was switched on and off alternately. As indicated by the blue dashed line, the short-circuit current is around 380 nA.

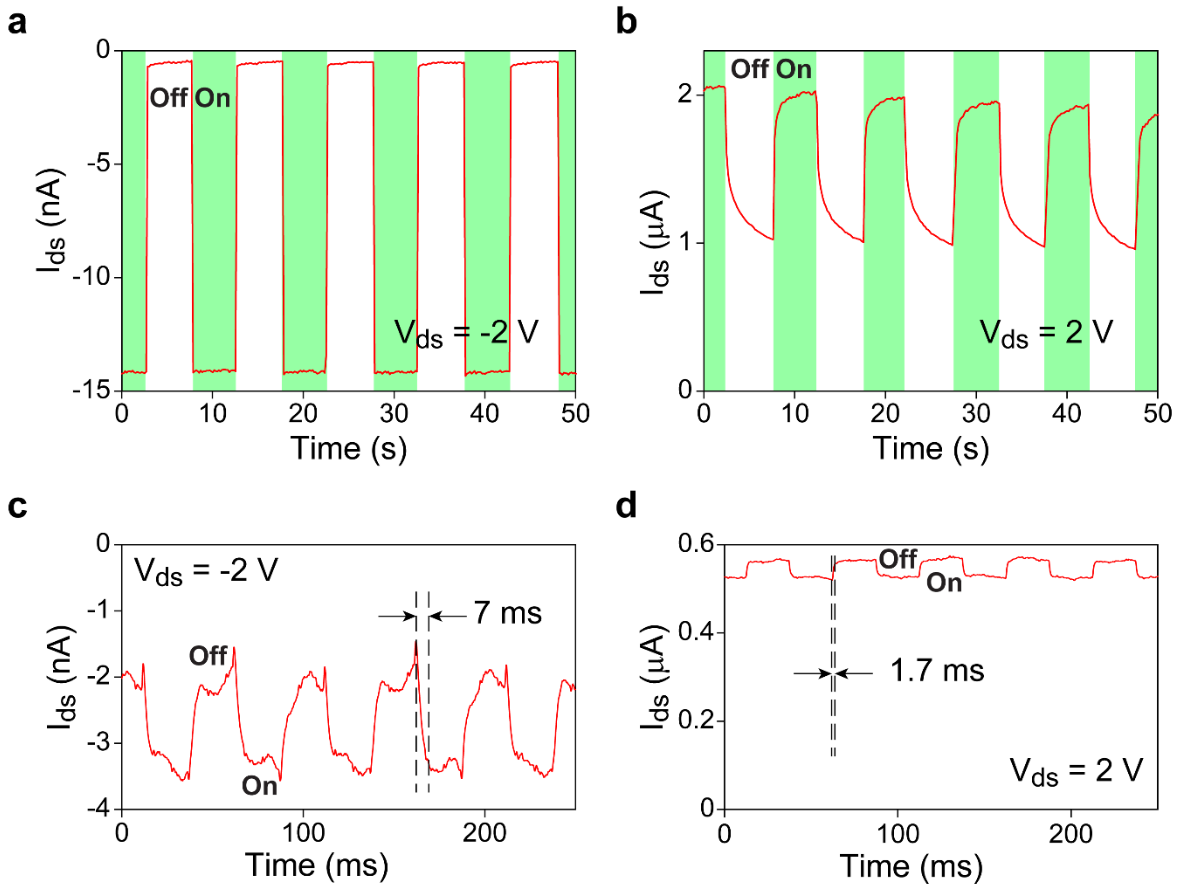

Figure S14. Transient photoresponse of InSe/$NbTe_2$ heterostructure device. (a, b) Time-dependent I_{ds} measured under reverse (a) and forward (b) bias. The illumination of 532 nm laser is manually switched On/Off, and gate voltage of V_{gate} = 0 V is applied for this measurement. (c, d) Response of the device under the illumination of a 520 nm laser switched On/Off with a frequency of 20 Hz, which is implemented with an optical chopper. Gate voltage of V_{gate} = +80 V is applied for this measurement. It's obvious that the response time of the InSe/$NbTe_2$ heterostructure device is < 10 ms.

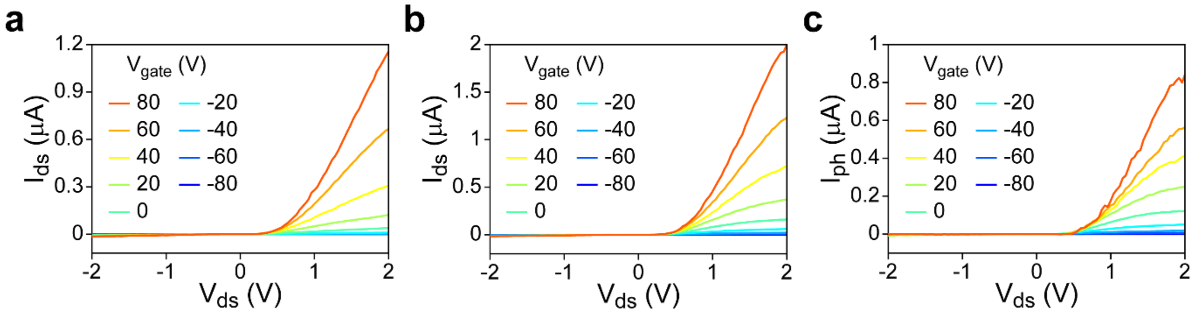

Figure S15. Photoresponse to 532 nm laser illumination with 10 nW power. (a) Output I_{ds} - V_{ds} curves measured with various V_{gate} in dark condition. (b) Output I_{ds} - V_{ds} curves when the 10 nW laser illumination mainly focused at pure InSe area in the channel of InSe/$NbTe_2$ heterostructure device. (c) Photocurrent I_{ph} calculated with the data in (a) and (b).

Based on the data shown above, the highest photoresponsivity is obtained at V_{ds} = 2 V and V_{gate} = 80 V, where I_{ph}/Power = 0.84 μA/10 nW = 84 A/W.

Table S1. Photoresponsivity of typical InSe-based photodetectors to the light illumination with specific wavelengths.

**Material(s)**	**Wavelength**	**Photoresponsivity (A/W)**	**Ref.**
InSe	532 nm	0.0347	3
InSe	532 nm	~ 6	4
InSe	370 nm	27	5
InSe	450 nm	12.3	6
InSe-Te	400 nm	0.45	7
InSe-GaTe	532 nm	200	8
InSe-$NbTe_2$	532 nm	84	This work

Table S2. Performance of InSe-based photodetectors with electrodes of various materials.

**Material of contact electrodes**	**Wavelength**	**Power or power density**	**Photoresponse**	**Ref.**
Graphene	633 nm	10 nW	~10 A/W	9
Few-layer graphene	850 nm	1 mW/cm^{-2}	~500 A/W	10
Al	543 nm	8.8 mW/cm^{-2}	~5 nA	11
MXene	855 nm	1 nW	10^4 A/W	12
Au and Ag	532 nm	10 nW	~50 A/W	13
Au	532 nm	10 nW	84 A/W	This work

References

1. Erdogan, H.; Kirby, R. D. Raman Spectrum and Lattice Dynamics of $NbTe_2$. *Solid State Commun.* **1989,** *70*, 713-715.

2. Jin, C.; Ma, E. Y.; Karni, O.; Regan, E. C.; Wang, F.; Heinz, T. F. Ultrafast Dynamics in van der Waals Heterostructures. *Nat. Nanotechnol.* **2018,** *13*, 994-1003.

3. Lei, S.; Ge, L.; Najmaei, S.; George, A.; Kappera, R.; Lou, J.; Chhowalla, M.; Yamaguchi, H.; Gupta, G.; Vajtai, R.; Mohite, A. D.; Ajayan, P. M. Evolution of the Electronic Band Structure and Efficient Photo-Detection in Atomic Layers of InSe. *ACS Nano* **2014,** *8*, 1263-1272.

4. Zhou, J.; Shi, J.; Zeng, Q.; Chen, Y.; Niu, L.; Liu, F.; Yu, T.; Suenaga, K.; Liu, X.; Lin, J.; Liu, Z. InSe Monolayer: Synthesis, Structure and Ultra-High Second-Harmonic Generation. *2D Mater.* **2018,** *5,* 025019.

5. Yang, Z.; Jie, W.; Mak, C.-H.; Lin, S.; Lin, H.; Yang, X.; Yan, F.; Lau, S. P.; Hao, J. Wafer-Scale Synthesis of High-Quality Semiconducting Two-Dimensional Layered InSe with Broadband Photoresponse. *ACS Nano* **2017,** *11*, 4225-4236.

6. Tamalampudi, S. R.; Lu, Y.-Y.; U, R. K.; Sankar, R.; Liao, C.-D.; B, K. M.; Cheng, C.-H.; Chou, F. C.; Chen, Y.-T. High Performance and Bendable Few-Layered InSe Photodetectors with Broad Spectral Response. *Nano Lett.* **2014,** *14*, 2800-2806.

7. Qin, F.; Gao, F.; Dai, M.; Hu, Y.; Yu, M.; Wang, L.; Feng, W.; Li, B.; Hu, P. Multilayer InSe-Te van der Waals Heterostructures with an Ultrahigh Rectification Ratio and Ultrasensitive Photoresponse. *ACS Appl. Mater. Interfaces* **2020,** *12*, 37313-37319.

8. Qi, T.; Gong, Y.; Li, A.; Ma, X.; Wang, P.; Huang, R.; Liu, C.; Sakidja, R.; Wu, J. Z.; Chen, R.; Zhang, L. Interlayer Transition in a vdW Heterostructure toward Ultrahigh Detectivity Shortwave Infrared Photodetectors. *Adv. Funct. Mater.* **2020,** *30*, 1905687.

9. Mudd, G. W.; Svatek, S. A.; Hague, L.; Makarovsky, O.; Kudrynskyi, Z. R.; Mellor, C. J.; Beton, P. H.; Eaves, L.; Novoselov, K. S.; Kovalyuk, Z. D.; Vdovin, E. E.; Marsden, A. J.; Wilson, N. R.; Patanè, A. High Broad-Band Photoresponsivity of Mechanically Formed InSe-Graphene van der Waals Heterostructures. *Adv. Mater.* **2015,** *27*, 3760-3766.

10. Jang, H.; Seok, Y.; Choi, Y.; Cho, S.-H.; Watanabe, K.; Taniguchi, T.; Lee, K. High-Performance Near-Infrared Photodetectors Based on Surface-Doped InSe. *Adv. Funct. Mater.* **2021,** *31*, 2006788.

11. Lei, S.; Wen, F.; Ge, L.; Najmaei, S.; George, A.; Gong, Y.; Gao, W.; Jin, Z.; Li, B.; Lou, J.; Kono, J.; Vajtai, R.; Ajayan, P.; Halas, N. J. An Atomically Layered InSe Avalanche Photodetector. *Nano Lett.* **2015,** *15,* 3048-3055.

12. Yang, Y.; Jeon, J.; Park, J.-H.; Jeong, M. S.; Lee, B. H.; Hwang, E.; Lee, S. Plasmonic Transition Metal Carbide Electrodes for High-Performance InSe Photodetectors. *ACS Nano* **2019,** *13*, 8804-8810.

13. Hu, S.; Zhang, Q.; Luo, X.; Zhang, X.; Wang, T.; Cheng, Y.; Jie, W.; Zhao, J.; Mei, T.; Gan, X. Au-InSe van der Waals Schottky Junctions with Ultralow Reverse Current and High Photosensitivity. *Nanoscale* **2020,** *12*, 4094-4100.
